# Genetic Influences on the Developing Young Brain and Risk for Neuropsychiatric Disorders

**DOI:** 10.1016/j.biopsych.2023.01.013

**Published:** 2023-05-15

**Authors:** Ann M. Alex, Claudia Buss, Elysia Poggi Davis, Gustavo de los Campos, Kirsten A. Donald, Damien A. Fair, Nadine Gaab, Wei Gao, John H. Gilmore, Jessica B. Girault, Karen Grewen, Nynke A. Groenewold, Benjamin L. Hankin, Jonathan Ipser, Shreya Kapoor, Pilyoung Kim, Weili Lin, Shan Luo, Elizabeth S. Norton, Thomas G. O’Connor, Joseph Piven, Anqi Qiu, Jerod M. Rasmussen, Michael A. Skeide, Dan J. Stein, Martin A. Styner, Paul M. Thompson, Laurie Wakschlag, Rebecca Knickmeyer, for the ENIGMA ORIGINs group

**Affiliations:** a Institute for Quantitative Health Sciences and Engineering, Michigan State University, East Lansing, Michigan; b Charité Universitätsmedizin Berlin, corporate member of Freie Universität Berlin and Humboldt-Universität zu Berlin, Department of Medical Psychology, Berlin, Germany; c Department of Pediatrics, University of California Irvine, Irvine, California; d Development, Health and Disease Research Program, University of California Irvine, Irvine, California; e Department of Psychology, University of Denver, Denver, Colorado; f Departments of Epidemiology & Biostatistics, Michigan State University, East Lansing, Michigan; g Department of Statistics & Probability, Michigan State University, East Lansing, Michigan; h Division of Developmental Paediatrics, Department of Paediatrics and Child Health, Red Cross War Memorial Children’s Hospital, University of Cape Town, Cape Town, South Africa; i Neuroscience Institute, University of Cape Town, Cape Town, South Africa; j Masonic Institute for the Developing Brain, University of Minnesota Medical School, Minneapolis, Minnesota; k Institute of Child Development, College of Education and Human Development, University of Minnesota, Minneapolis, Minnesota; l Department of Pediatrics, University of Minnesota Medical School, Minneapolis, Minnesota; m Harvard Graduate School of Education, Harvard University, Cambridge, Massachusetts; n Cedars–Sinai Biomedical Imaging Research Institute, Los Angeles, California; o Departments of Biomedical Sciences and Imaging, Cedars–Sinai Medical Center, Los Angeles, California; p Department of Psychiatry, University of North Carolina, Chapel Hill, Chapel Hill, North Carolina; q Carolina Institute for Developmental Disabilities, University of North Carolina at Chapel Hill, Carrboro, North Carolina; r South African Medical Research Council Unit on Child and Adolescent Health, University of Cape Town, Cape Town, South Africa; s Department of Paediatrics and Child Health, University of Cape Town, Faculty of Health Sciences, Cape Town, South Africa; t Psychology Department, University of Illinois Urbana,-Champaign, Illinois; u Research Group Learning in Early Childhood, Max Planck Institute for Human Cognitive and Brain Sciences, Leipzig, Germany; v Department of Psychology, University of Denver, Denver, Colorado; w Department of Radiology, University of North Carolina at Chapel Hill, Chapel Hill, North Carolina; x Department of Medicine, Keck School of Medicine of the University of Southern California, Los Angeles, California; y Department of Psychology, University of Southern California, Los Angeles, California; z Center for Endocrinology, Diabetes and Metabolism, Children’s Hospital Los Angeles, Los Angeles, California; aa Roxelyn and Richard Pepper Department of Communication Sciences and Disorders, Northwestern University, Evanston, Illinois; bb Department of Medical Social Sciences and Institute for Innovations in Developmental Sciences, Feinberg School of Medicine, Northwestern University, Chicago, Illinois; cc Departments of Psychiatry, Psychology, Neuroscience, Obstetrics and Gynecology, University of Rochester, Rochester, New York; dd Department of Biomedical Engineering, National University of Singapore, Singapore; ee NUS (Suzhou) Research Institute, National University of Singapore, China; ff the Institute for Health, National University of Singapore, Singapore; gg School of Computer Engineering and Science, Shanghai University, Shanghai, China; hh Institute of Data Science, National University of Singapore, Singapore; ii Department of Biomedical Engineering, the Johns Hopkins University, Baltimore, Maryland; jj South African Medical Research Council Unit on Risk and Resilience in Mental Disorders, Department of Psychiatry, University of Cape Town, Cape Town, South Africa; kk Department of Computer Science, University of North Carolina at Chapel Hill, Chapel Hill, North Carolina; ll Imaging Genetics Center, Stevens Neuroimaging & Informatics Institute, Keck School of Medicine of University of the Sunshine Coast, Marina del Rey, California; mm Department of Pediatrics and Human Development, Michigan State University, East Lansing, Michigan

**Keywords:** Childhood, Genetics, Imaging, Infant, Magnetic resonance imaging, Pediatric

## Abstract

Imaging genetics provides an opportunity to discern associations between genetic variants and brain imaging phenotypes. Historically, the field has focused on adults and adolescents; very few imaging genetics studies have focused on brain development in infancy and early childhood (from birth to age 6 years). This is an important knowledge gap because developmental changes in the brain during the prenatal and early postnatal period are regulated by dynamic gene expression patterns that likely play an important role in establishing an individual’s risk for later psychiatric illness and neurodevelopmental disabilities. In this review, we summarize findings from imaging genetics studies spanning from early infancy to early childhood, with a focus on studies examining genetic risk for neuropsychiatric disorders. We also introduce the Organization for Imaging Genomics in Infancy (ORIGINs), a working group of the ENIGMA (Enhancing NeuroImaging Genetics through Meta-Analysis) consortium, which was established to facilitate large-scale imaging genetics studies in infancy and early childhood.

Imaging genetics reveals considerable information about genetic influences on structural and functional imaging phenotypes (1, 2, 3), but until recently focused largely on the adolescent or adult human brain (4,5). This is an important limitation because the most dynamic phase of human brain development is from embryonic life through early childhood (6) (Figure 1). Disrupted gene expression in this period can produce lifelong changes in brain morphology and function. Even common genetic variations may affect early neurodevelopmental processes, thereby increasing risk for psychiatric conditions later in life (7). These effects may be detectable in early life via neuroimaging, thereby providing opportunities for identifying at-risk populations in infancy for primary prevention and the development of interventions to adjust adverse trajectories earlier in the clinical sequence. In this paper, we review empirical evidence underlying this hypothesis focusing on magnetic resonance imaging (MRI). Studies using ultrasound (8,9) and studies integrating imaging and epigenetic data (10, 11, 12, 13) also provide insights into how genes influence the developing young brain but are beyond the scope of this review. First, we describe the heritability of brain imaging phenotypes in early life. Then, we discuss candidate gene studies of brain structure, function, and connectivity. Next, we review studies characterizing associations between psychiatric risk genes and brain phenotypes in early life using polygenic scores. Then, we discuss genome-wide studies on brain imaging phenotypes in early childhood. Finally, we introduce ORIGINs (the Organization for Imaging Genomics in Infancy), a working group of the ENIGMA (Enhancing NeuroImaging Genetics through Meta-Analysis) consortium, which was established to facilitate large-scale imaging genetics studies in infancy and early childhood.

**Figure 1:**
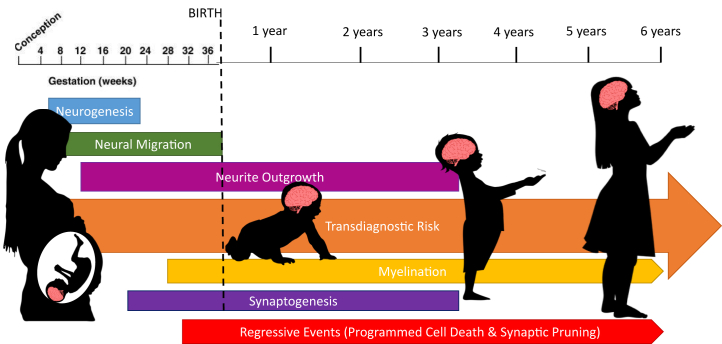
Early neurodevelopment is a sensitive period for accumulating transdiagnostic risk for psychiatric disorders. Neurodevelopment in the human brain begins from approximately 2 weeks after conception. Neurogenesis and neural migration are primarily prenatal processes, and neurite outgrowth is minimal after 4 years of age. Myelination and synaptic pruning continue beyond 6 years of age (indicated by the small arrows). Genetic influences on these various processes contribute to psychiatric risk, which accumulates across development and may not manifest until later in life (large orange arrow).

## Heritability

Twin studies have revealed that many brain phenotypes are heritable in early infancy. Genetic effects explain around 85% of the variance in global white matter volume (WMV) and 56% of the variance in global gray matter volume (GMV), at around 1 month of age (14), while heritability of head size is negligible (15). This contrasts with studies of older children and adults where heritabilities greater than 80% are reported for all 3 phenotypes (global WMV, GMV, and head size) (16, 17, 18). Heritability estimates for global cortical surface area (SA) are high in early infancy (78%), while estimates for global cortical thickness (CT) are lower (29%), with significant genetic overlap between the two (19). This differs from adults, among whom SA and CT are both highly heritable (89% and 81%, respectively), with distinct genetic factors contributing to each measure (20, 21, 22). White matter microstructure is moderately heritable in early life, with 30% to 60% of the variability in mean fractional anisotropy (FA) linked to genetic variation and similar estimates for other diffusivity indices (23,24). In adults, estimates for FA range from 72% to 88% (25). Despite large variations in heritability estimates across individual tracts, a single latent measure of white matter microstructure accounts for a great deal of heritable variation in neonates (50%) (26). Similarly, individual differences in regional CT and SA appear to be driven by a common set of genetic factors influencing cortical structure at the global level (19). In both cases, the pattern of results mirrors temporal changes in gene expression which show strong spatial differences in fetal but not postnatal development (27,28). Finally, genetic effects on resting-state functional MRI phenotypes have been observed during the first 2 years of life. Gao *et al.* (29) reported modest genetic effects on within-network connectivity in neonates, with 3 visual networks and the right frontoparietal network demonstrating above-average effects. At age 1 year, the most heritable networks were the bilateral frontoparietal networks, the salience network, and 2 visual networks. At age 2 years, genetic effects were the strongest for the auditory network. However, genetic effects were not as strong as those reported in adolescents and adults (30, 31, 32). Genetic effects on between-network connectivity are also minimal in neonates (33). Intergenerational transmission of imaging phenotypes has been reported and likely reflects a combination of genetic, epigenetic, and environmental effects (34, 35, 36). One such study examined the intergenerational transmission from mothers to their 5-year-old children and reported significant effects on sulcal phenotypes in the right frontal and parietal cortices (35).

A recurring theme across studies is that heritability is higher in adulthood than in infancy. This might appear paradoxical because interindividual variation in environmental exposures increases with age, but similar patterns are observed for IQ, where increasing heritability during development is called the Wilson effect (37). The Wilson effect is thought to arise from gene-environment correlations that increase with age. In other words, babies and young children have environments thrust upon them, but as they age, they select, modify, and create environments that are correlated with their genetic predispositions (38). Alternatively, higher heritability observed at later ages could reflect stronger heritability of postnatal processes such as myelination and shifts in proportions of white versus gray matter across development. Determining whether a genetic amplification model applies to neuroimaging phenotypes will require large-scale longitudinal studies that address gene-environment interplay across the life span. The ENIGMA plasticity working group has begun tackling this question. Using 5 longitudinal twin cohorts, they demonstrated that rates of brain change are heritable, and heritability estimates of change rates were higher in adults than in children (39). They subsequently identified variants involved in structural brain changes via a genome-wide association study (GWAS) (40). However, their studies did not include infants or toddlers.

### Candidate Gene Approaches

Traditional candidate gene studies test for associations between phenotypic outcomes and variation within specific genes selected for suspected roles in organ development or physiology. In imaging genetics, selection is often based on hypothesized involvement in psychiatric disease. The first candidate gene study of brain imaging phenotypes in neonates focused on global and local brain tissue volumes and several genes with known roles in brain development and putative links to psychiatric disease including *DISC1*, *COMT*, *NRG1*, *ESR1*, and *BDNF* (41). Many reported effects mirrored findings in adults; others were unique to infancy. For the *BDNF* Val/Met polymorphism, Met+ neonates had decreased volumes in regions of the right occipital cortex, left hippocampus, parahippocampus, fusiform gyrus, and inferior temporal gyrus and increased volumes in the motor and somatosensory cortex (41). We highlight this result because a recent study partially replicated the original findings. Specifically, Kawasaki *et al.* (42) reported that Met+ neonates had significantly smaller relative hippocampal volumes.

The largest traditional candidate gene study to include children under 6 years of age focused on Klotho, a gene linked to age-related decline. A significant interaction between Klotho allele status (rs9536314) and age was observed for total brain volume and total GMV, with KLS-VS heterozygotes having larger volumes in early childhood but not in later childhood/adolescence. Among girls, KL-VS heterozygotes had less WMV than noncarriers, whereas among boys, heterozygotes had greater WMV than noncarriers. No effects were significant in a replication cohort that did not include children younger than 6 years of age (43), supporting the importance of conducting imaging genetics studies in early life to unveil effects that have been found to be absent in older cohorts.

In addition to age, genetic effects on neurodevelopment may vary based on factors such as prematurity and family history. For example, Krishnan *et al.* (44) hypothesized that polymorphisms in *DLG4* would moderate responses to perinatal inflammation and their impact on white matter microstructure based on gene network analysis of the microglial transcriptomic response to injury in mouse models and complementary, data-driven analysis of protein-protein interactions, transcription factors, and human brain gene expression. The team discovered a specific variant in *DLG4* (rs17203281) associated with FA in preterm individuals in 2 independent cohorts. Van Steenwinckel *et al.* (45) adopted a similar approach, identifying key genes and gene networks in animal models of neuroinflammation-induced hypomyelination and then testing for associations in preterm infants. The researchers revealed that Wnt pathway genes were collectively associated with cerebral structural connectivity. In addition, a study of 13 candidate genes revealed that *ARVCF*, previously linked to schizophrenia, and *FADS2*, previously linked to intelligence, were associated with white matter FA in preterm infants (46). These studies highlight the importance of considering potential interactions between genetic variation and early-life environmental exposures given that neither *DLG4* nor the Wnt pathway genes would be expected to impact diffusion tensor imaging phenotypes in the absence of perinatal inflammation. With regard to family history, Douet *et al.* (47) reported that the effects of variants in *ERBB4* differed in children with and without a family history of schizophrenia and/or bipolar disorder. The TT variant for rs7598440 had more pronounced effects on age-related changes (3–20 years) in CT and SA in children with a family history; these children showed steeper increases in frontal and temporal SA in both early and late childhood.

**Figure 2:**
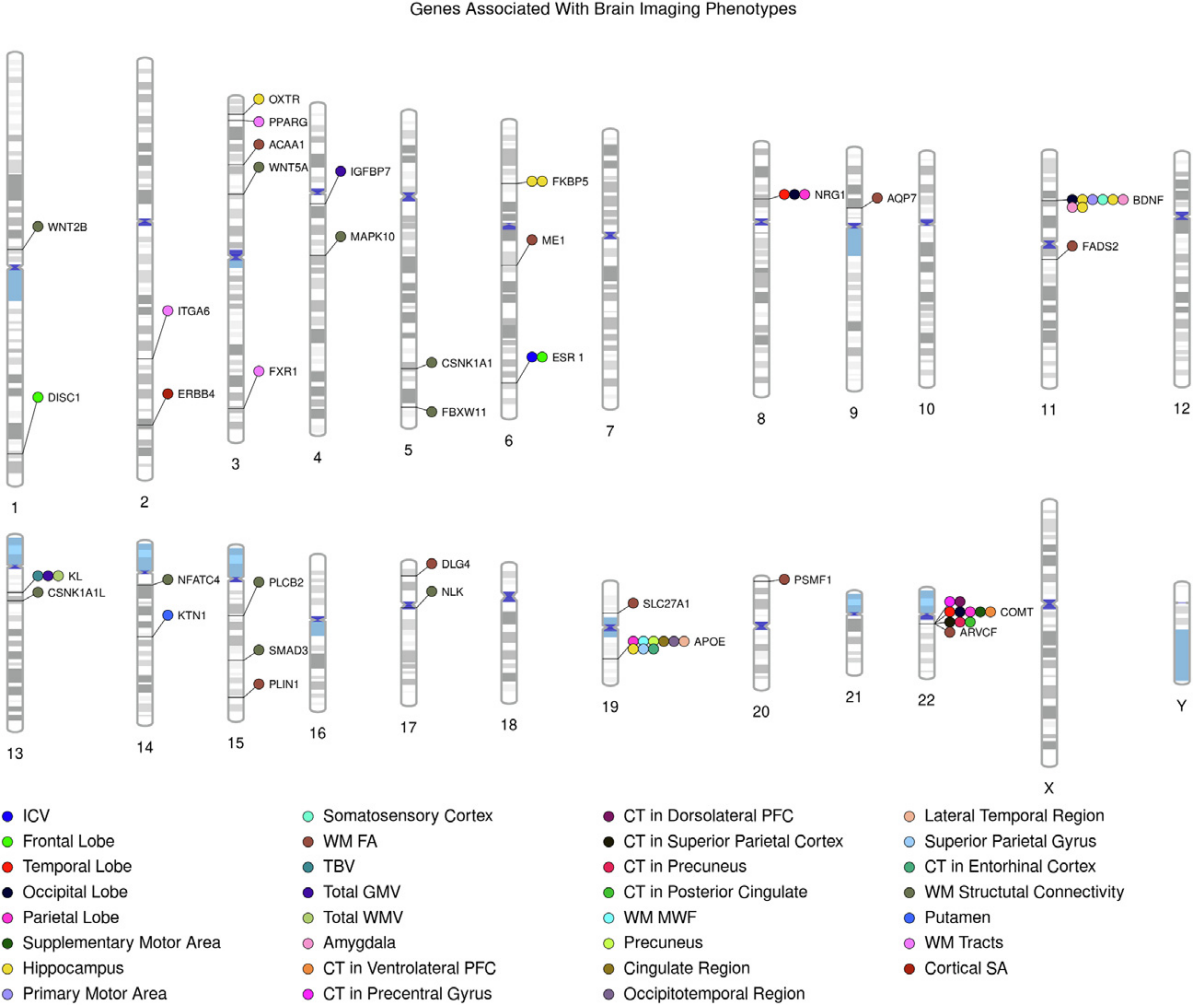
PhenoGram of genes associated with brain imaging phenotypes in infants and young children from candidate gene studies and genome-wide association studies. CT, cortical thickness; FA, fractional anisotropy; GMV, gray matter volume; ICV, intracranial volume; MWF, myelin water fraction; PFC, prefrontal cortex; SA, surface area; TBV, total brain volume; WM, white matter; WMV, white matter volume.

Several studies explicitly tested for gene-environment interactions using the candidate gene approach. *COMT* single nucleotide polymorphisms (SNPs) moderated the association between antenatal maternal anxiety and prefrontal and parietal CT in neonates (48). The *BDNF* genotype (Val66Met) moderates associations between methylation patterns and neonatal hippocampal and amygdala volumes (49). *FKBP5*, which regulates the hypothalamic-pituitary-adrenal axis, moderates the association between antenatal maternal depressive symptoms and neonatal right hippocampal volume (50). For oxytocin receptor (*OXTR*) gene variant rs53576, a sex-specific main effect was seen for neonatal hippocampal volume. Left hippocampal volumes were larger in GG-homozygotes than A-allele carriers in boys only. Prenatal maternal anxiety interacted with genotype in both sexes: higher maternal anxiety was associated with larger hippocampal volumes in A-allele carriers (51). Additional details on these studies are found in Table 1. A graphical representation (PhenoGram) (52) of genes and associated phenotypes is given in Figure 2.

**Table 1:** Candidate Gene Studies of Imaging Phenotypes in Infancy and Childhood

Article	Participants, *N*	Age Group	Ancestry	Gene/SNP-Brain Phenotype Association	Findings
Studies on Genetic Effects					
Knickmeyer *et al.*, 2014 (41)	272	Neonates (gestational age at MRI: 261–433 days)	Maternal ethnicity—White	*ESR1* (rs9340799)—ICV; *DISC1* (rs821616), *COMT*, *NRG1*, *APOE*, *ESR1* (rs9340799), and *BDNF*—GMV	Associations in *DISC1* and *COMT* mirrored findings in adults.
Dean *et al.*, 2014 (56)	162	2- to 25-month-old infants	Not reported	*APOE* ε4 allele—↓ MWF and GMV in precuneus, posterior/middle cingulate, lateral temporal, and medial occipitotemporal regions. *APOE* ε4 allele—↑ MWF and GMV in extensive frontal regions	Infant *APOE* ε4 allele carriers had lower white matter MWF and GMV measurements than noncarriers in areas preferentially affected by Alzheimer’s disease.
Boardman *et al.*, 2014 (46)	83 preterm infants	Neonates (postmenstrual age 23 + 2 to 32 + 6 weeks)	Multiancestry	*ARVCF* (rs2518824) and *FADS2* (rs174576)—white matter FA	–
Douet *et al.*, 2015 (47)	971 (PING study)	3–20 years	Multiancestry	*ERBB4* (rs7598440)—cortical structures	In the full sample, children with the TT genotype had smaller SA in the occipital and temporal lobes at ages <5 years. When stratifying by family history of schizophrenia and/or bipolar disorder, TT children showed steeper increases in frontal SA in early childhood.
Chang *et al.*, 2016 (58)	1187 (PING study)	3–20 years	Multiancestry	*APOE* (ε2ε4—↓ hippocampus; ε4ε4—↓ hippocampal FA; ε3ε4—↑ medial orbitofrontal cortical areas)	The ε4ε4 and ε2ε4 genotypes may negatively influence brain development and brain aging at the extremes of age.
Krishnan *et al.*, 2017 (44)	Preterm infants (cohort 1: *n* = 70; cohort 2 [ePRIME study]: *n* = 271)	Cohort 1—mean postmenstrual age at scan 40 + 3 weeks; cohort 2—mean postmenstrual age at scan 42 + 4 weeks	Multiancestry	*DLG4* (rs17203281)—FA	*DLG4* (rs17203281) was associated with structural white matter changes.
Van Steenwinckel *et al.*, 2019 (45)	290 preterm infants (ePRIME study)	Gestational age of 38.29–58.28 weeks	Multiancestry	*NFATC4*, *CSNK1A1*, *MAPK10*, *WNT2B*, *SMAD3*, *FBXW11*, *NLK*, *CSNK1A1L*, *PLCB2,* and *WNT5A*—white matter structural connectivity	Genomic variation in the Wnt pathway is associated with the levels of connectivity found in their brains.
De Vries *et al.*, 2020 (43)	1387 (PING study)	3–21 years	Multiancestry	Klotho allele KL-VS; KL-CS × age interaction—TBV, TGMV; Kl-VS × sex—TWMV	A replication in a cohort of 2306 children age 6–12 years (Generation R sample) showed no significant associations. KL-VS’s influence may depend on age and sex.
Remer *et al.*, 2020 (57)	223	2–68 months	Multiancestry	*APOE* ε4 carriers—MWF	ε4 carriers—significant MWF trajectory differences in multiple neuroanatomical locations
Kawasaki *et al.*, 2021 (42)	66	Newborn infants (37.9–47.6 postmenstrual weeks)	Multiancestry	*BDNF*-Val66Met variant—hippocampi, amygdalae, TWMV	Met + group—↓ hippocampi, amygdalae, age-dependent declines in % total WMVs, slower age-dependent declines in total brain volumes
Cullen *et al.*, 2022 (59)	208 (dHCP study)	0–6 weeks	European	rs945270 (intergenic locus downstream of the kinectin 1 [*KTN1*] gene)—putamen volume	Greater number of C alleles associated with larger volume

Studies on Interaction Between Genetic and Environmental Effects					
Qiu *et al.*, 2015 (48)	146 (GUSTO cohort)	Neonates (5–17 days)	Asian	*COMT*—cortical thickness	*COMT* SNPs (val158met, rs737865 and rs165599)—role in moderating the relationship of antenatal maternal anxiety with dorsolateral prefrontal and parietal cortical thickness in neonates
Chen *et al.*, 2015 (49)	237 (GUSTO)	Neonates (4–17 days)	Asian	*BDNF* (Val66Met)—hippocampus, amygdala	*BDNF* (Val66Met)—regulate the sensitivity of the methylome with differential effects on amygdala and hippocampal volume
Wang *et al.*, 2017 (50)	164 Mother-offspring dyads (GUSTO)	Neonates (5–14 days)	Asian	*FKBP5*—hippocampus	17 SNPs in the *FKBP5* gene showed significant interaction effects with antenatal maternal depressive symptoms on right hippocampal volume.
Acosta *et al.*, 2021 (51)	105	11–54 days old	European	*OXTR* rs53576 × sex—hippocampus	For *OXTR* SNP rs53576, in boys compared with girls, left hippocampal volumes were significantly larger in GG-homozygotes than A-allele carriers. Higher maternal anxiety was associated with larger hippocampal volumes in A-allele carriers than GG-homozygotes.

dHCP, developing Human Connectome Project; FA, fractional anisotropy; GMV, gray matter volume; GUSTO, Growing Up in Singapore Toward healthy Outcomes; ICV, intracranial volume; MRI, magnetic resonance imaging; MWF, myelin water fraction; PING, Pediatric Imaging, Neurocognition, and Genetics consortium; SA, surface area; SNP, single nucleotide polymorphism; TBV, total brain volume; TGMV, total GMV; TWMV, total white matter volume.

Interestingly, imaging genetics studies of infants and young children began at a time when microarray genotyping began making large-scale genotyping practical. The GWAS era quickly highlighted weaknesses in the traditional candidate gene approach. Well-powered GWASs failed to support involvement of many traditional candidate genes in psychiatric disorders. This may partly reflect addressable methodological weaknesses including failure to control for population stratification and thereby increasing the risk of false-positive associations due to differences in ancestry. However, the key disadvantage of the candidate gene approach is likely poor candidate selection, given the inadequacy of current knowledge about underlying biological processes. Subsequent meta-analyses of candidate gene studies relevant to psychiatry, including imaging genetics studies conducted in older populations, revealed poor replicability, false-positive associations, overestimation of effect sizes, and publication bias (53). While the imaging genetics literature for infants and young children is not extensive enough to allow meta-analyses, existing studies likely have similar weaknesses.

One promising approach to addressing these problems is to focus future studies on variants that are robustly associated with mental and neurological disorders or adult brain imaging phenotypes in large-scale GWASs. The ϵ4 allele of the apolipoprotein E (*APOE*) gene meets this criterion. Not only is ϵ4 the strongest known risk variant for Alzheimer’s disease, it also has well-documented effects on brain structure and cognition in healthy individuals (54,55). In neonates, *APOE* ϵ3ϵ4 heterozygotes have significantly lower volumes in temporal regions, compared with ϵ3 homozygotes, and lower volume in the frontal and parietal lobes. ϵ3ϵ4 heterozygotes have significantly greater volumes in specific parietal, frontal, and occipital areas (41). Infant ε4 carriers have lower white matter myelin water fraction (MWF) and GMV measurements in the precuneus, posterior/middle cingulate, lateral temporal, and medial occipitotemporal regions, areas which are preferentially affected by Alzheimer’s disease, and greater MWF and GMV measurements in frontal regions (56). Decreased myelin in ε4 carriers in the corticospinal tract, the splenium of the corpus callosum, and frontal white matter, observed in the previous study, was also reported in a longitudinal analysis from the same group in which children were followed from birth to age 5.5 years (57). Regions in which ε4 carriers had greater MWF early on had a decreased rate of MWF development until age 5.5 years, allowing noncarriers to catch up and surpass ε4 carriers at around 3 years of age. In another study, age-related changes in brain structures and cognition were observed to vary depending on genotype, with the smallest hippocampi in ε2ε4 children, the lowest hippocampal FA in younger ε4ε4 children, the largest medial orbitofrontal cortical areas in ε3ε4 children, and age-dependent thinning of entorhinal cortex in ε4ε4 children (58). All these studies suggest that Alzheimer’s disease is a neurodevelopmental disorder as well as a neurodegenerative one.

Six SNPs robustly associated with subcortical volumes in adult GWASs have recently been tested for effects in neonates. An association between rs945270 (an intergenic locus downstream of the kinectin 1 [*KTN1*] gene) and putamen volume was reported, suggesting that at least some variants have detectable effects across the life span (59).

A significant challenge when performing a candidate gene study informed by existing GWASs is how to prioritize genes for follow-up. Fortunately, an increasing array of in silico tools for searching GWAS literature and performing functional characterization of variants can assist with this task (60, 61, 62). Another challenge is that candidate gene studies informed by GWASs still focus on only a few selected genes/polymorphisms which account for only a fraction of variants involved in psychiatric risk. One approach to overcoming this challenge is to use polygenic risk scores.

### Polygenic Risk Score Approaches

Polygenic risk scores (PRSs) estimate an individual’s susceptibility to a complex trait based on prior GWAS summary statistics (63). Efforts such as the Psychiatric Genomics Consortium have produced many well-powered GWASs of psychiatric and neurodevelopmental conditions including schizophrenia, major depressive disorder (MDD), bipolar disorder, attention-deficit/hyperactivity disorder, and autism spectrum disorder (ASD) (64). Summary statistics are often freely available, and researchers can obtain individual data from controlled-access repositories. By examining associations between PRSs and neuroimaging phenotypes measured in infancy and early childhood, researchers are clarifying how genetic risk for these conditions manifests in early life, thereby providing new insights into the etiology of psychiatric and neurodevelopmental disorders. This is a key step in identifying individuals who may benefit from early intervention.

One of the first studies to use this approach in early life was conducted by Xia *et al.* (65), who found that PRSs for schizophrenia and ASD were not associated with neonatal total brain volume. Like Xia *et al.* (65), Cullen *et al.* (59) did not observe associations between neonatal volumes and PRSs for schizophrenia. However, PRSs for schizophrenia were negatively associated with regional GMV and WMV and total WMV in neonates in a different study (66). PRSs for ASD were associated with greater CT and reduced white matter connectivity in children (3 to ∼14 years) (67). In preterm infants, a PRS for 5 conditions (ASD, attention-deficit/hyperactivity disorder, bipolar disorder, MDD, and schizophrenia) predicted reduced volume of the lentiform nucleus, which plays a key role in motor control, cognition, and emotion (68). The authors hypothesized that genetic risk for psychiatric disorders increased vulnerability to abnormal lentiform development in the context of perinatal stress associated with preterm birth but did not include term infants for comparison. Other studies have used PRSs to probe relationships between early-life adversity, genetic risk, and neurodevelopment. Ursini *et al.* (69) used transcriptomic data to create placental genomic risk scores (PlacGRSs) for schizophrenia. PlacGRSs were calculated like traditional PRSs, but only used markers in genes highly expressed in placenta and differentially expressed in placentae from complicated, compared with normal, pregnancies. PlacGRS was negatively associated with neonatal brain volume in children with perinatal complications, especially in boys. No significant associations were observed for PlacGRSs and nonplacental GRSs for other disorders and traits associated with early-life complications, suggesting that the link between placental biology, genetic risk, perinatal environmental risk, and early brain development outcomes is relatively unique to schizophrenia.

Another important perinatal stressor is maternal depression. Many studies report associations of maternal depressive symptoms with neuroimaging outcomes in early life, but it is unclear whether such associations represent causal effects or arise from genetic confounding. PRSs can be used to test independent effects of maternal depressive symptoms and genetic risk for MDD as well as their interaction. Qiu *et al.* (70), the first to apply this approach, reported significant interactions between PRSs for MDD and antenatal maternal depressive symptoms for right amygdala volume in Asian (GUSTO [Growing Up in Singapore Towards healthy Outcomes]) and U.S. neonates. However, the direction of the effect differed across cohorts. In Finland, Acosta *et al.* found patterns similar to those found in the U.S. cohort (71). However, the interaction became nonsignificant after correction for multiple comparisons. The Finnish team also investigated associations of an MDD PRS with infant striatal volumes and found sex-specific effects: the MDD PRS was positively associated with caudate volumes in boys but negatively associated with caudate volumes in girls (72). They did not observe significant interaction effects of the PRS with prenatal maternal depressive symptoms for any dorsal striatal volumes.

PRSs can also be used to understand how molecular pathways shape individual differences in neurodevelopment. For example, a PRS for serum testosterone was recently found to be positively associated with total SA development in female infants (73). Researchers interested in this application of PRSs may implement expression-based PRSs (ePRSs) rather than traditional PRSs. ePRSs integrate genotype data with transcriptomic data to predict expression levels of a particular gene or gene network. Morgunova *et al.* (74) used this approach to investigate relationships between a coexpression network of the *DCC* gene, which is robustly associated with multiple psychiatric conditions, and total brain volume in both neonates and older children. Higher ePRSs for the *DCC* coexpression network were associated with larger brain volumes. A study from GUSTO investigated how genes involved in inflammation interact with maternal depression to shape neonatal brain morphology. They created separate ePRSs for 22 cytokine and chemokine genes expressed in fetal brain and found that ePRSs for *TNFRSF19*, *IL17RB*, *BMPR1B*, *IL1RAP*, and *CXCR4* moderated the impact of maternal depression on specific subcortical volumes and regional CT (75). Using longitudinal data from the same cohort, investigators revealed an age-dependent involvement for transmembrane receptor (TGF-β) variants in moderating effects of prenatal maternal depressive symptoms on amygdala volume (76).

Finally, PRSs have been used to investigate how genetic variants linked to adult and adolescent brain morphology influence early brain development. Xia *et al.* (65) found that PRSs for WMV and GMV in adolescence showed positive associations with neonatal WMV and GMV, respectively, although the overall proportion of variation explained was low. Morgunova *et al.* (74) calculated PRSs for brain volume using data from UK Biobank, ENIGMA, CHARGE (Cohorts for Heart and Aging Research in Genomic Epidemiology), and the Early Growth Genetics Consortium. These PRSs did not predict brain volume in their neonate and school-age community cohorts. The first large-scale GWAS of adult intracranial volume tested whether a polygenic score generated from 7 genome-wide significant loci predicted head growth in children of European ancestry who were followed prenatally until 6 years of age (77). The investigators found an age-dependent effect in which the PRS became more predictive in older children, suggesting that adult brain volume is strongly shaped by genetic influences operating in early childhood. Cullen *et al.* (59) found robust associations between PRSs for adult brainstem, hippocampus, putamen, and thalamus volumes and neonatal volumes, suggesting some stability across the life course.

Reviewed studies highlight the potential of PRS-based investigations of neuroimaging phenotypes in infancy and early childhood (Table 2). They also reveal the importance of considering effects of sex and ancestry. A major limitation of this approach is the lack of sufficiently powered GWASs conducted in non-European populations. European ancestry GWASs do not transfer well to other ancestries and can lead to unpredictable biases (78). Another limitation of PRS studies is that they are based on existing GWASs and hence limited by the power of current datasets. In other words, PRS-based studies, like candidate gene studies, are constrained by current biological knowledge. To fully understand how DNA variants influence brain development, well-powered GWASs of infants and young children encompassing multiple ancestries are needed.

**Table 2:** PRS Studies of Imaging Phenotypes in Infancy and Childhood

Article	Participants, *N*	Age Group	Ancestry	PRS	Findings
Studies on Main Genetic Effects					
Xia *et al.*, 2017 (65)	561	6–161 days	Multiancestry	Polygenic scores for GM and WM from adolescent cohort; polygenic scores for ICV from adolescent and adult cohort	Adolescent WM and GM scores showed positive associations with neonatal WM and GM; adult polygenic scores for ICV did not predict neonatal ICV
				PRS for schizophrenia and ASD	PRS did not predict global brain volumes.
Cullen *et al.*, 2019 (68)	194 preterm infants	Mean postmenstrual age at scan 42.6 weeks	Multiancestry	PRS from meta-analysis of genome-wide SNP data for 5 psychiatric disorders (ASD, ADHD, bipolar disorder, MDD, and schizophrenia)	↑PRS—↓ lentiform volume in the mixed ancestral cohort and a European subsample
Khundrakpam *et al.*, 2020 (67)	391 (PING study)	3–21 years	Multiancestry	PRS for ASD	↑ PRS for ASD—↑ cortical thickness for a large age span starting from 3 years up to ∼14 years in several cortical regions localized in the bilateral precentral gyri and the left hemispheric postcentral gyrus and precuneus, ↓WM connectivity between the frontal and parietal regions
Morgunova *et al.*, 2021 (74)	142	Neonates (27 ± 13 days)	Multiancestry	ePRS was created based on the *DCC* coexpression gene network in the PFC.	↑ ePRS—↑ total brain volume (GM and WM, adjusted by ICV)
Alex *et al.*, 2021 (73)	430	Birth–2 years	European	PRS for serum testosterone	↑ PRS—↑ SA development over time in female infants
Cullen *et al.*, 2022 (59)	208	0–6 weeks	European	GPSs for adult subcortical brain volumes	Neonatal volumes of the hippocampus, brainstem, putamen, and thalamus associated with adult GPS
				GPSs for psychiatric disorders ASD, ADHD, schizophrenia, bipolar disorder, MDD, and cross-disorder (including 8 psychiatric disorders: anorexia nervosa, ADHD, ASD, bipolar disorder, MDD, obsessive-compulsive disorder, schizophrenia, and Tourette syndrome)	None of the neonatal brain volumes showed an association with psychiatric GPS.
Le *et al.*, 2022 (66)	257	Postmenstrual age at scan 38–45 weeks	Preliminary analysis: European; secondary; European and Asian	PRS for schizophrenia	↑PRS—↓ right frontal lobe WM, ↓GM and WM superior temporal gyrus volumes and ↓total white matter volume

Studies on Interaction Between Genetic and Environmental Risk					
Qiu *et al.*, 2017 (70)	168 (GUSTO) and 85 (US) mother-infant dyads	Neonates: GUSTO (5–14 days), US (postconceptual age at the MRI visit 43.02 ± 2.1 weeks)	GUSTO—Asian; US—mixed ancestry	GPRSMDD	A significant interaction was observed between antenatal maternal depressive symptoms and infant GPRSMDD on right hippocampal volume in the Asian cohort and right amygdala volume in both cohorts. A significant interaction was observed between SES and infant GPRSMDD on right amygdala and hippocampal volumes and shapes in the Asian cohort.
Wang *et al.*, 2017 (50)	164 Mother-offspring dyads (GUSTO)	Neonates (5–14 days)	Asian	A genetic risk score was calculated for individual neonates by summing the number of minor alleles of 19 *FKBP5* SNPs.	Neonates with a genetic risk score less than or equal to the median showed a positive association between antenatal maternal depressive symptoms and right hippocampal volume. Neonates with a genetic risk score greater than the median showed a negative association between antenatal maternal depressive symptoms and right hippocampal volume.
Acosta *et al.*, 2020 (71)	105	11–54 days old	European	PRS-MDD	A nonsignificant interaction effect was observed between PRS-MDD and prenatal maternal depressive symptoms on right amygdala volume.
Acosta *et al.*, 2020 (72)	105	11–54 days old	European	PRS-MDD	No significant interaction effects of PRS-MDD with prenatal maternal depressive symptoms were found for infant dorsal striatal volumes. PRS-MDD was more positively associated with caudate volumes in boys compared with girls.
Wu *et al.*, 2020 (75)	161 mother-child dyads (GUSTO)	Neonates (5–14 days)	Asian	A GES was calculated for individuals by summing the number of minor alleles across the SNPs of the gene that were highly correlated with its expression level according to the existing eQTL database.	Positive associations of prenatal maternal depressive symptoms with the hippocampal volume, auditory and prefrontal cortical thickness in neonates high in GESs of the TNF, IL-1, IL-17, chemokine, and TGF family and receptors.
Ursini *et al.*, 2021 (69)	242	10–60 days	European	Fractionated genomic risk scores for schizophrenia based on placental gene expression loci (PlacGRSs)	↑PlacGRSs —↓ neonatal brain volume in singletons and offspring of multiple pregnancies with a history of early-life complications
Qiu *et al.*, 2021 (76)	162 (GUSTO)	Birth–6 years	Asian	A GES was calculated for individuals by summing the number of alleles across the SNPs of the gene that was correlated with TGF-βRI expression level according to the existing eQTL database.	In neonates with a high GES of *TGFBR1*, higher levels of prenatal maternal depressive symptoms were associated with a smaller right amygdala volume. In children with a low GES of *TGFBR1*, greater prenatal maternal depressive symptoms predicted greater left and right amygdala volumes at 6 years of age.

ADHD, attention-deficit/hyperactivity disorder; ASD, autism spectrum disorder; ePRS, expression-based polygenic risk score; eQTL, expression quantitative trait loci; GES, genetic expression score; GM, gray matter; GPRSMDD, genomic profile risk score for major depressive disorder; GPS, genome-wide polygenic score; GUSTO, Growing Up in Singapore Toward healthy Outcomes; ICV, intracranial volume; IL, interleukin; MDD, major depressive disorder; MRI, magnetic resonance imaging; PING, Pediatric Imaging, Neurocognition, and Genetics consortium; PFC, prefrontal cortex; PlacGRS, placental genomic risk score; PRS, polygenic risk score; SA, surface area; SES, socioeconomic status; SNP, single nucleotide polymorphism; US, United States; WM, white matter.

### Genome-wide Association Studies

Hypothesis-free GWASs can identify new associations and overturn prior assumptions. However, GWASs of neuroimaging outcomes in infants and young children are very limited. The first GWAS of healthy infants identified several common variants associated with neonatal brain structure (65). An intronic SNP in *IGFBP7* was significantly associated with GMV. An intronic SNP in *WWOX* fell just short of genome-wide significance for WMV. Many top associations tagged transcriptional regulators expressed during brain development (*KLF13*, *LMCD1*, *TOX3*, and *TBX4*). The investigators also compared their results to large-scale neuroimaging GWASs in adolescents and adults and concluded that genetic determinants of global brain volumes are highly distinct at different ages. In a subsequent GWAS of white matter microstructure in neonates, an intronic SNP in *PSMF1* was significantly associated with a tractography-based factor capturing shared variation in FA across 44 white matter bundles (26). Additional loci nearing genome-wide significance were in or near genes with roles in axon growth and guidance, fasciculation, and myelination including *B3GAT1*, *TENM2*, *NFATC1*, and *MAP3K13*. The above studies are the first of their kind, and replication is crucial. Furthermore, these studies were not large enough to generate stable SNP-wise heritability estimates or evaluate genomic correlations between infant neuroimaging phenotypes and psychiatric disorders.

A smaller study in preterm individuals used genome-wide data and pathway-based and network-based approaches (79). The peroxisome proliferator-activated receptor signaling pathway was found to have a role in white matter development, with 5 genes implicated (*AQP7*, *ME1*, *PLIN1*, *SLC27A1*, and *ACAA1*). This inspired the team to examine the peroxisome proliferator-activated receptor pathway in a larger cohort of preterm children. Using machine learning analysis, they uncovered 3 genes associated with cerebral connectivity (*PPARG*, *ITGA6*, and *FXR1*) (80). GWASs are summarized in Table 3 and included in the Figure 2 PhenoGram (52). Gene functions and associated neurological phenotypes/conditions for candidate genes and genes identified via GWASs are provided in Table 4.

**Table 3:** Genome-wide Association Studies of Imaging Phenotypes in Infancy and Childhood

Article	Participants, *N*	Age Group	Ancestry	Findings
Krishnan *et al.*, 2016 (79)	72 preterm infants	Gestational age 23 + 2 to 32 + 6 weeks	Multiancestry	Identified significant role for lipid pathways and PPAR signaling in influencing development of white matter in preterm infants. Five genes were found to be highly associated with the phenotype: *AQP7*, *ME1*, *PLIN1*, *SLC27A1*, and *ACAA1*.
Krishnan *et al.*, 2017 (80)	272 preterm infants	Gestational age 42 weeks + 4 days	Multiancestry	*PPARG* (6 SNPs), *ITGA6* (4 SNPs), *FXR1* (2 SNPs) are associated with preterm cerebral endophenotype, particularly insular cortex
Xia *et al.*, 2017 (65)	561	6–161 days	Multiancestry	An intronic^a^ SNP in *IGFBP7* (rs114518130) achieved genome-wide significance for gray matter volume. An intronic SNP in *WWOX* (rs10514437) neared genome-wide significance for white matter volume.
Zhang *et al.*, 2021 (26)	471	Neonates (days post conception 293.4 ± 16.6)	Multiancestry	An intronic SNP in the gene *PSMF1* was significant for a tractography-based factor that captured shared variation in fractional anisotropy across 44 white matter bundles.

PPAR, peroxisome proliferator-activated receptor; SNP, single nucleotide polymorphism.

a Intronic SNPs are located in a segment of a DNA or RNA molecule which does not code for proteins and interrupts the sequence of genes.

**Table 4:** Brain Imaging Phenotype Associated Genes, Their Functions, and Associated Neurologic Phenotypes/Disorders

Gene	Symbol	Function	Associated Neurologic Phenotype(s)/Disorder(s)
Acetyl-CoA Acyltransferase 1	*ACAA1*	Involved in neuronal growth and myelinogenesis (87)	Alzheimer’s disease (88)
Apolipoprotein E	*APOE*	Facilitates the transfer of cholesterol and phospholipid between cells, key role in neuronal development, brain plasticity, and repair (89)	Alzheimer’s disease, schizophrenia (41)
Aquaporin 7	*AQP7*	Allows movement of water, glycerol, and urea across cell membranes^a^	–
Armadillo Repeat Gene Deleted in Velocardiofacial Syndrome	*ARVCF*	Modulates neural cell-cell adhesion and migration (46)	Schizophrenia (46)
Brain-Derived Neurotrophic Factor	*BDNF*	Regulates cell survival, axonal outgrowth, dendritic growth, and synaptic plasticity (90)	Depression, bipolar disorder, schizophrenia, anxiety, autism, ADHD, substance abuse, eating disorders, Alzheimer’s disease (41)
Casein Kinase 1, Alpha 1	*CSNK1A1*	Suppressor of Wnt/β-catenin signaling^a^	Schizophrenia (91)
Casein Kinase 1, Alpha 1-like	*CSNK1A1L*	Involved in negative regulation of canonical Wnt signaling pathway and peptidyl-serine phosphorylation^a^	–
Catechol-O-Methyltransferase	*COMT*	Degrades dopamine and other catecholamines (92)	Schizophrenia (41)
Discs Large MAGUK Scaffold Protein 4	*DLG4*	Synapse structure and development (44)	Intellectual disability, epilepsy, autism spectrum disorder, schizophrenia (44,93)
Disrupted in Schizophrenia 1	*DISC1*	Neural migration, neurite outgrowth, and dendritic arborization (94)	Schizophrenia, bipolar disorder, autism, depression (41)
Erb-B2 Receptor Tyrosine Kinase 4	*ERBB4*	Role in neurodevelopment such as glial and neuronal migration, myelination, excitatory neuronal receptor expression, and the onset of puberty (47)	Schizophrenia, bipolar disorder (47)
Estrogen Receptor 1	*ESR1*	Mediates estrogen effects on synaptogenesis, growth factor production, and responses to oxidative stress (95)	Anxiety, depression, schizophrenia, Alzheimer’s disease (41)
Fatty Acid Desaturase 2	*FADS2*	Essential for neurogenesis, neurotransmission, and protection from oxidative stress (46)	Interact with early dietary exposures to influence childhood IQ (46)
F-Box and WD Repeat Domain Containing 11	*FBXW11*	Involved in ubiquitination and proteasomal degradation (96)	Autism spectrum disorder (96)
FK506-Binding Protein 5	*FKBP5*	Transcriptional regulation of the HPA axis (50)	Depression, PTSD (50)
Fragile X Mental Retardation, Autosomal Homolog 1	*FXR1*	Levels of FXR1 are important for parvalbumin interneurons (97)	Schizophrenia, bipolar disorder (98)
Insulin-like Growth Factor-Binding Protein 7	*IGFBP7*	Regulation of availability of IGFs^a^	Learning and memory (99)
Integrin Subunit Alpha 6	*ITGA6*	Involved in insulin-like growth factor 1 signaling (80)	Schizophrenia (100)
Kinectin 1	*KTN1*	Encodes the protein kinectin, a receptor that allows vesicle binding to kinesin and is involved in organelle transport (59)	ADHD (59)
Klotho	*KL*	Health and survival (43)	Cognition (43)
Malic Enzyme 1	*ME1*	Sex-specific gene regulation in the offspring, key regulator of a T2DM-specific gene expression network (101,102)	–
Mitogen-Activated Protein Kinase 10	*MAPK10*	Neuronal proliferation, differentiation, migration, and programmed cell death^a^	Cognition (103)
Nemo-like Kinase	*NLK*	Positive effector of the noncanonical Wnt signaling pathway and negative regulator of the canonical Wnt/beta-catenin signaling pathway^a^	–
Neuregulin 1	*NRG1*	Mediate cell-cell interactions in the brain and other organs, neuronal migration and specification, oligodendrocyte differentiation and myelination, regulation of acetylcholine, and expression of GABA receptors (104)	Schizophrenia, bipolar disorder (41)
Nuclear Factor of Activated T-Cells, Cytoplasmic 4	*NFATC4*	Hippocampal plasticity, axonal growth, neuronal survival, and apoptosis (105)	Spatial memory^a^
Oxytocin Receptor	*OXTR*	Receptor for oxytocin (51)	Depression, autism, eating disorder (51)
Perilipin 1	*PLIN1*	Regulates droplet formation in lipopolysaccharide-stimulated microglia (106)	–
Peroxisome Proliferator-Activated Receptor Gamma	*PPARG*	Regulator of adipocyte differentiation^a^	Schizophrenia (107)
Phospholipase C, Beta 2	*PLCB2*	Catalyzes the hydrolysis of phosphatidylinositol 4,5-bisphosphate^a^	Schizophrenia (108)
Proteasome Inhibitor Subunit 1	*PSMF1*	Inhibits activation of the 26S proteasome, a multicatalytic proteinase complex that may play a role in developmental axonal pruning and synaptic plasticity (109)	–
SMAD Family Member 3	*SMAD3*	Involved in regulating inflammatory responses (110)	Alzheimer’s disease (110), cognition (111)
Solute Carrier Family 27 (Fatty Acid Transporter), Member 1	*SLC27A1*	Involved in fatty acid transport across the blood-brain barrier (112)	–
Wingless-Type MMTV Integration Site Family, Member 2B	*WNT2B*	Regulation of cell growth and differentiation^a^	Bipolar disorder (113)
Wingless-Type MMTV Integration Site Family, Member 5A	*WNT5A*	Essential role in regulating developmental pathways during embryogenesis^a^	Schizophrenia (114), memory (115)

ADHD, attention-deficit/hyperactivity disorder; GABA, gamma-aminobutyric acid; HPA, hypothalamic-pituitary-adrenal; IGF, insulin-like growth factor; PTSD, posttraumatic stress disorder; T2DM, type 2 diabetes mellitus.

a GeneCards (116).

General strengths and limitations of GWASs have been reviewed in detail elsewhere (81). In terms of GWASs in infants and young children, the primary limitations of existing studies include their being 1) underpowered due to small samples, 2) mostly cross-sectional rather than longitudinal, 3) only focused on neonates and preterm infants, and 4) the fact that individuals of non-European ancestry constitute such a small proportion of the total samples.

### Rigor and Reproducibility

When we consider the rigor and reproducibility of published imaging genetics studies in infants and young children, insufficient power and sample size are significant concerns. Sample sizes for early childhood imaging genetics studies are low, with a mean of 365 for candidate gene studies (median = 216), a mean of 225 for PRS-based studies (median = 168), and a mean of 344 for GWASs (median = 371.5). Consequently, existing studies are powered to detect variants with large effect sizes. It is likely that most variants impacting infant brain imaging phenotypes will explain between 0.1% and 1% of the variance, like other complex traits (82). Furthermore, small samples can produce unstable results, and homogenous sampling can generate statistical inferences that do not represent the overall population. Independent replication is essential to validate results and improve estimation of effects but is currently rare due to difficulties recruiting and scanning large groups of infants.

To improve rigor and reproducibility and fully understand how DNA variants influence brain development in infancy and early childhood across diverse populations and the implications for future research and clinical care, large longitudinal studies are needed. ORIGINs was founded to facilitate such work.

## Organization for Imaging Genomics in Infancy

ORIGINs includes investigators from different centers around the world (16 sites, 19 cohorts, 5 countries) who are engaged in neuroimaging research in infancy and early childhood. Our goal is to determine how genetic and environmental factors influence development of brain morphometry, anatomical and functional connectivity, and cognitive and emotional function from birth to age 6 years. In 2020, we received National Institutes of Health funding to create the largest-ever imaging genomics dataset focused on infancy and early childhood. In subsequent sections, we briefly describe who will be included in this dataset, what is being measured, and our data analysis plans.

### Participants

Participants will include approximately 6809 children (birth to 6 years of age) participating in neuroimaging studies of early brain development at the University of North Carolina Chapel Hill, University of California Irvine, Max Planck Institute for Human Cognitive and Brain Sciences, Rhode Island Hospital, Northwestern University, University of Denver, University of Rochester, Magee-Womens Hospital of the University of Pittsburgh Medical Center, University of Cape Town, Boston’s Children Hospital/Harvard University, University of Minnesota, University of Washington, Washington University in St. Louis, King’s College London, and National University of Singapore. Eight cohorts have completed initial data collection and 11 are actively scanning. We estimate that 51% of the sample will be female, 49% will be male, 0.05% will be American Indian, Alaska Natives, Native Hawaiians, or Pacific Islanders, 11% will be Asian, 20% will be Black, 6% will be more than one ancestry, and 62% will be White. Individuals identifying as Hispanic/Latinx are expected to make up 15% of the cohort.

### Data Measurements

#### Demographic and Medical History

Health history and demographic information of participants were provided by parents or guardians and/or extracted from medical records. The information includes birth outcomes (gestational age, birth weight), sex, socioeconomic factors (maternal education, total family income), and family history of medical and neuropsychiatric disorders.

#### Genomic Data

Most participating sites have used/are using saliva samples for DNA extraction. Two cohorts (Drakenstein Child Health Study and GUSTO) used umbilical cord and venous blood specimens. To harmonize data across genotyping platforms, we will impute genomes to a common set of SNPs using the Michigan Imputation Server (83). See the Supplement for details on harmonization and genotyping platforms used by each cohort.

#### Behavioral Assessments

In a subset of participants (∼3800), we will examine 3 behavioral traits—impulsivity/distractibility, anxiety, and aggressive behavior—that can be reliably measured in very young children and are relevant to multiple psychiatric disorders. Behavioral traits will be measured using age-appropriate versions of the Child Behavior Checklist (84) and the Behavior Assessment System for Children, Second Edition (85,86).

#### Image Acquisition and Quality Control

3T Siemens scanners (Allegra, Tim Trio, Verio, Skyra, and Prisma) and comparable sequences (Tables S3–S6) were used with all cohorts except for the dHCP (developing Human Connectome Project) and GUSTO. dHCP data were acquired on a Philips Achieva, and newborn T2 structural MRI acquisition for GUSTO was on a 1.5T General Electric scanner. To ensure consistent processing across datasets with the same tools and appropriately standardized parameter settings, all structural, diffusion, and functional connectivity data will be processed at a central site. T1 and T2 structural MRI, diffusion tensor imaging, and resting-state functional MRI acquisition parameters, platform, and harmonization pipeline for each site are detailed in the Supplement.

#### Data Analysis Plan

**Figure 3:**
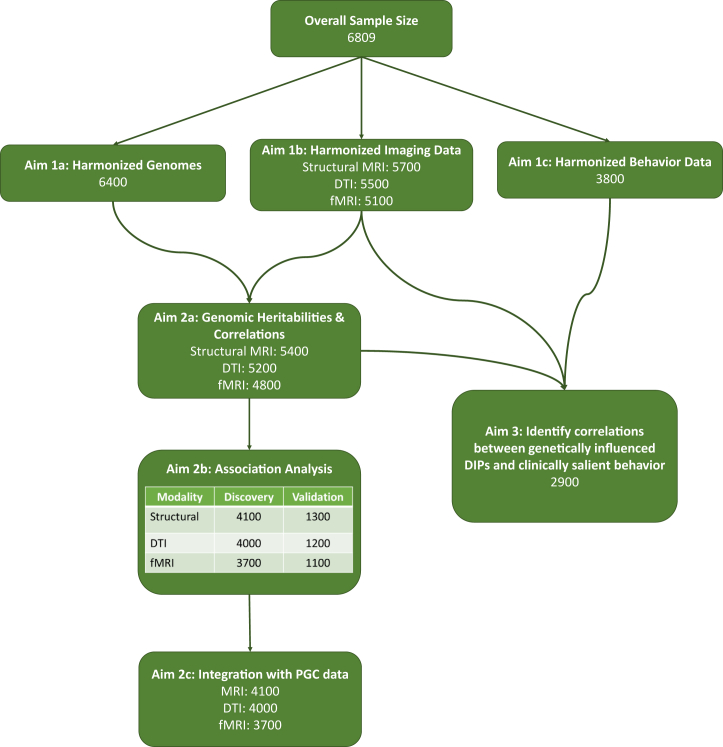
Data analysis plan for the ORIGINs (Organization for Imaging Genomics in Infancy). DIP, developmental imaging phenotype; DTI, diffusion tensor imaging; fMRI, functional magnetic resonance imaging; PGC, Psychiatric Genomics Consortium.

Extracted neuroimaging measures will be analyzed using nonlinear growth models. Growth model parameters, which we refer to as “developmental imaging phenotypes,” will be used to test effects of genetic variants on structural brain development and connectivity using a multivariate GWAS approach. Canonical correlation analysis will be used to identify association patterns between genetically influenced neurodevelopmental traits and clinically salient behaviors. The data analysis and data sharing plan is detailed in the Supplement. A schematic for data analysis is provided as Figure 3.

## Conclusions

Imaging genetics studies of infants and young children have provided evidence that variants associated with psychiatric disorders influence early neurodevelopment both independently and through interactions with environmental factors. In addition, GWASs of neonates and preterm infants have revealed new genes, variants, and molecular pathways implicated in brain development. However, most findings have not been independently replicated. Existing studies, regardless of design, are relatively small and do not encompass diverse ancestries. The ORIGINs initiative is addressing these limitations by creating and harmonizing the largest and most diverse imaging genomics dataset focused on infancy and early childhood to date. This dataset will help reveal how genetic risk for psychiatric disease manifests across infancy and early childhood, in terms of brain structure and function, and assist in early identification of at-risk individuals. Ultimately, identifying genes and molecular pathways associated with early neuroimaging phenotypes could lead to the development of novel prophylactics against complex psychiatric illness.
