# Supplementary material for: Genetic Influences on the Developing Young Brain and Risk for Neuropsychiatric Disorders
Source: Biol Psychiatry. 2023 May 15;93(10):905–20. doi: 10.1016/j.biopsych.2023.01.013 (PMC10136952; doi:10.1016/j.biopsych.2023.01.013)
Supplement: Supplementary Materials and Tables [file mmc1.pdf]

SUPPLEMENTARY INFORMATION

Genetic Influences on the Developing Young Brain and Risk for Neuropsychiatric Disorders

Alex *et al.*

Supplementary Material

ORIGINs will assemble a large and well-characterized imaging genetics dataset through collaborations with world-leaders in infant neuroimaging. This dataset will include information from approximately 6809 infants and young children (birth to 6 years of age) representing 19 parent cohorts. The details on the participant number and study design for each cohort is given in Supplementary Table 1.

Supplementary Table 1: Participant number and study design for each ORIGINs site

Cohort	Site/Study	Participants (N)	Study Design
1	UNC Early Brain Development Study (EBDS)	866	Longitudinal
2	Growing Up in Singapore Towards Healthy Outcomes (GUSTO), Singapore	500	Longitudinal
3	Infant Brain Imaging Study (IBIS)	210	Longitudinal
4	UNC Grewen 1	173	Longitudinal
5	University of California Irvine (UCI)	142	Longitudinal
6	Max Planck Institute for Human Cognitive and Brain Sciences, Germany	136	Cross-sectional
7	UNC Multi-visit Advanced Pediatric Brain Imaging Study (MAP)	94	Cross-sectional
8	UNC Gut Microbiome and Infant Anxiety (GMIA)	149	Longitudinal
9	Developing Human Connectome Project (King's College London, Imperial College London and Oxford University)	950	Longitudinal
10	Baby Connectome Project	500	Accelerated longitudinal
11	Drakenstein Child Health Study (DCHS), South Africa	290	Longitudinal
12	UNC Grewen 2	275	Longitudinal
13	Northwestern Promoting Healthy Brain Project (PHBP)	100	Longitudinal
14	Harvard University/Boston's Children Hospital	184	Longitudinal
15	Northwestern When to Worry (W2W)	100	Longitudinal
16	University of Denver Care Project	300	Longitudinal
17	University of Denver Prenatal Pathways (PP)	200	Cross-sectional
18	Rhode Island Hospital	1070	Accelerated longitudinal
19	Rochester-Magee	570	Longitudinal

Genomic Data: As ORIGINs includes sites with existing data and ongoing recruitment, the platform for genotyping varies and is described in Supplementary Table 2. To harmonize data generated by different genotyping platforms, we will impute genomes to a common set of SNPs using the Michigan Imputation Server. To analyze ancestry within each data set, we will use self-reported ethnicity confirmed with principal component analyses. The classifications are based on the NIH reporting criteria for racial and ethnic categories (American Indian or Alaska Native, Asian, Black or African American, Hispanic or Latino, Native Hawaiian or Other Pacific Islander, and White). The Black participants from our South African cohort (DCHS) are of African ancestry, and the participants from our Singapore cohort (GUSTO) are of Asian ancestry. The Black and Asian participants from the US cohorts are African Americans and Asian Americans respectively. The black participants from the UK cohort might include Caribbean and African ancestry. We will cluster our samples using the SNP confirmed ancestries (expected groups include Caucasians, African American, Hispanics, East Asian, and admixed) and impute each of the subgroups with a separate library (HRC for Caucasians, CAPA for African Americans, TopMed for Hispanics and admixed individuals, and 1000 Genomes for East Asians). We will apply standard quality control (QC) on the genotypes (matching reported and genetic sex, removing SNPs with low calling rates) before imputation. For each data set, we will retain only imputed SNPs with predicted R2 of at least 80%, which yields an actual R2 over 90% for both African Americans and Hispanics.

Supplementary Table 2: Genotyping platforms used by each ORIGINs site

Site	Genotyping platform
Early Brain Development Study (EBDS), UNC(1)	Affymetrix Axiom® Genome-Wide LAT and Exome arrays
Growing Up in Singapore Towards healthy Outcomes (GUSTO), Singapore(2)	Illumina Omni express arrays and Illumina Exome arrays
Infant Brain Imaging Study (IBIS)	Illumina HumanOmni5 and Exome arrays
University of California, Irvine(3)	Illumina OmniExpress arrays
Drakenstein Child Health Study (DCHS), Cape Town(4,5)	Illumina PsychArray Beadchip
Rhode Island Cohort	Custom chip from Illumina*
Developing Human Connectome project	Illumina 5m chip
All other sites	Infinium Global Screening Array BeadChip

*This chip was based on the MEGA chip with additional content from NeuroX.

Image Analysis: All structural, diffusion MR images, and functional connectivity data will be processed at a central site (UNC: structural and diffusion MRI, Cedars-Sinai: resting state functional MRI) to ensure consistent processing across all imaging datasets with the same tools and appropriately standardized parameter settings. Outlined below are the planned image analysis methods for each modality to address site-specific differences in acquisition parameters (Supplementary Tables 3-6).

High-Resolution MRI: To adjust for site-related resolution differences, scans will be up- or down-sampled into a common $1mm^3$ isotropic resolution, corrected for intensity inhomogeneity via N4(6), and rigidly co-registered to ICBM space using age-specific pediatric MNI templates(7). Skull-stripping will then remove non-brain regions keeping both brain tissue regions (cerebral and cerebellar) as well as all sub-arachnoidal cerebrospinal fluid (CSF) spaces. Tissue segmentation and subcortical region segmentation will be carried out using automatic, multi-atlas tissue

segmentation via the MultiSeg tool developed for the full pediatric age range (8–10). Cortical surface reconstruction and quantification of cortical thickness, surface area, and sulcal folding is performed with NeoCIVET for neonates and CIVET for older subjects(11,12) employing the cortical Destrieux parcellation(13). All processing steps will be visually assessed for appropriate quality. In case of failure, the dataset will either be excluded or reprocessed with manual registration initialization. No manual editing of the label/segmentation results is performed. If available, both T1-weighted and T2-weighted MRI will be employed jointly in the above processing. In case T2-weighted MRIs are not available for a cohort, then the whole cohort will be processed only with the T1-weighted MRIs. Similarly, when T1-weighted MRIs are not available for a cohort, then the whole cohort will be processed only with the T2-weighted MRIs.

Supplementary Table 3: T1 Structural MRI Acquisition Parameters for each ORIGINs site

Cohort	Site	Platform	T1 Scan types	TR	TE	Resolution
1	UNC-EBDS	Allegra	FLASH	15	7	0.9x0.9x1.0
			MP-Rage	1820	4.38	1.0x0.9x0.9
			MP-Rage	1820-1900	4.38	1x1x1
		TIM Trio	MP-Rage	1820-1900	3.74-3.75	1x1x1
		Prisma	MP-Rage	2400	2.22	0.8x0.8x0.8
2	Singapore	Skyra	MP-RAGE	2000	2.08	1x1x1
3	IBIS	TIM Trio	MP-Rage	2400	2.16	1x1x1
4	UNC-Grewen 1	Allegra	MP-Rage	1820	3.75	1x1x1
		TIM Trio	MP-Rage	1820	3.75	1x1x1
5	UCI	TIM Trio	MP-Rage	2400	3.16	1x1x1
6	MPI	TIM Trio	MP-Rage	5000	2.82	1.3x1.3x1.3
7	UNC-MAP	Allegra	MP-Rage	1820	4.38	1x1x1
		TIM Trio	MP-Rage	1820	3.75	1x1x1
8	UNC-GMIA	TIM Trio	MP-Rage	1900	3.89	0.8x0.8x0.8
		Prisma	MP-Rage	2400	2.22	0.8x0.8x0.8
9	dHCP	Achieva	TSE	4795	8.7	0.8x0.8x0.8
10	BCP (UNC/UMN)	Prisma	MP-Rage	2400	2.22	0.8x0.8x0.8
11	DCHS Cape Town	Allegra	MP-RAGE	2530	1.64,3.5,5.36, 7.22,9.08	1x1x1
		Skyra	MP-RAGE	2530	1.64,3.5,5.36, 7.22,9.08	1x1x1
12	UNC-Grewen 2	Prisma	MP-Rage	2400	2.22	0.8x0.8x0.8
13	Northwestern-PHPB	Prisma	MP-RAGE	2400	3.19	0.8x0.8x0.8
14	Harvard	TIM Trio	mocoEMPRAGE	2270	1.45	1x1x1
15	Northwestern-W2W	Prisma	MP-RAGE	2400	3.19	0.8x0.8x0.8
16	Denver-Care	Skyra	MP-RAGE	1900	3.07	0.82x0.82x0.8
17	Denver-Kim	Prisma	MP-RAGE	2400	2.24	0.8x0.8x0.8
18	Rhode Island	TIM Trio	MP-Rage	1900	1.1	1.2x1.2x1.2
		Prisma	MP-Rage	1900	1.1	1.2x1.2x1.2
19	Rochester/Pitt	Skyra	MP-RAGE	2400	2.24	0.8x0.8x0.8

Supplementary Table 4: T2 Structural MRI Acquisition Parameters for each ORIGINs site

Cohort	Site	Platform	T2 Scan types	TR	TE	Resolution
1	UNC-EBDS	Allegra	TSE	5270-6200	119-124	1.25x1.25x1.95
			TSE	7380-7590	119	1.25x1.25x1.5
			TSE	6200	116	1.25x1.25x1.95
		TIM Trio	3D SPACE	3200	406	1x1x1
		Prisma	3D SPACE	3200	563	0.8x0.8x0.8
2	Singapore	1.5T GE	TSE	3500	110	1x1x2
3	IBIS	TIM Trio	FSE	3200	499	1x1x1
4	UNC-Grewen 1	Allegra	TSE	6200	116	1.3x1.3x1.5
		TIM Trio	TSE	6200	116	1.3x1.3x1.5
5	UCI	TIM Trio	TSE	3200	255	1x1x1
7	UNC-MAP	Allegra	TSE	5270	119	1.3x1.3x1.5
		TIM Trio	TSE	6200	116	1.3x1.3x1.5
8	UNC-GMIA	TIM Trio	3D SPACE	3200	406	0.8x0.8x0.8
		Prisma	3D SPACE	3200	563	0.8x0.8x0.8
9	dHCP	Achieva	TSE	12000	156	0.8x0.8x0.8
10	BCP (UNC/UMN)	Prisma	3D SPACE	3200	563	0.8x0.8x0.8
11	DCHS Cape Town	Allegra	3D SPACE	3500	354	1x1x1
		Skyra	3D SPACE	3500	354	1x1x1
12	UNC-Grewen 2	Prisma	3D SPACE	3200	563	0.8x0.8x0.8
13	Northwestern-PHPB	Prisma	3D SPACE	3200	563	0.8x0.8x0.8
15	Northwestern-W2W	Prisma	3D SPACE	3200	563	0.8x0.8x0.8
16	Denver-Care	Skyra	TSE	3200	408	0.86x0.86x0.8
17	Denver-PP	Prisma	TSE	3200	564	0.8x0.8x0.8
19	Rochester/Pitt	Skyra	TSE	3200	564	0.8x0.8x0.8

Diffusion Tensor Imaging (DTI): For DTI, the primary issues affecting harmonization are different numbers of unique gradient directions and b-values across sites. Synthetic simulations and living phantom studies show that beyond 30 directions, the number of unique gradient directions has limited effects on quantification of diffusion tensor properties(14–16). Even when comparing to directional data(17), a linear correction via site-specific nuisance covariates captures most of the inter-site variation(18), although we prefer and will use more sophisticated approaches such as ComBat(19). The same is true for inter-site variation due to different b-values if b-values range 700-1500 and analysis focuses on fractional anisotropy (FA), which is less sensitive to inter-site differences than other tensor measures such as axial (AD) and radial (RD) diffusivity(20). In order to reduce variability across sites, we will run tensor estimation excluding any information acquired at b values >1500. We will use atlas-based functional fiber profile analysis to analyze WM microstructure in 12 major fiber tracts(21). Automated QC will be performed by DTIPrep(22) and FSL (23) 6.0.8 including detection of slice-wise intensity changes and excessive motion artifacts, correction of susceptibility, motion and eddy current effects, and interpolation of outlier voxels (24). Diffusion images with excessive number of volumes with larger motion artifacts, missing or corrupted sections will be excluded from later analysis. Additional expert-guided QC will be performed with 3DSlicer(25). Our analysis framework is based on streamline tractography performed in an hierarchical unbiased average DTI tensor atlas(26,27). Tracts are parameterized by length to reveal diffusion properties as a function of location along the tracts, and functional statistical analysis methods(28–30) used to compute local statistics.

Supplementary Table 5: DTI Acquisition Parameters for each ORIGINs site

**Cohort**	**Site**	Platform	B-values	Gradient Directions	TR	TE	Resolution
1	UNC-EBDS	Allegra	b=0, b=1000*5 repetitions	6	5200	73	2x2x2
			b=0, b=1000	42	7680	82	2x2x2
		TIM Trio	b=0, b=1000	42	7200	83	2x2x2
		Prisma	b=0, b=1500, b=3000	79	3222	89.2	1.5x1.5x1.5
2	Singapore	Skyra	B=0, b=1000	30	8200	85	2x2x2
3	IBIS	TIM Trio	Variable (1000)<br>Variable (2000)	25<br>65	12800<br>11100	102<br>103	2x2x2
4	UNC-Grewen 1	Allegra	b=0, b=1000	42	7200	83	2x2x2
		TIM Trio	b=0, b=1000	42	7200	83	2x2x2
5	UCI	TIM Trio	b=0, b=1000	42	8900	83	2x2x2
6	MPI	TIM Trio	b=0, b=1000	60	8000	83	1.9x1.9x1.9
7	UNC-MAP	Allegra	b=0, b=1000	42	7680	82	2x2x2
8	UNC-GMIA	TIM Trio	b=0, b=300, b=700, b=2000	117	4000	90	2x2x2
		Prisma	b=0, b=1500, b=3000	79	3222	89.2	1.5x1.5x1.5
9	dHCP	Achieva	b=0, b=400, b=1000, b=2600	280	3800	90	1.5x1.5x1.5
10	BCP (UNC/UMN)	Prisma	b=0, b=1500, b=3000	79	3222	89.2	1.5x1.5x1.5
11	Cape Town	Allegra	b=0, b=1000	45	7900	90	1.6x1.4x1.3
		Skyra	b=0, b=1000	45	7900	90	1.6x1.4x1.3
12	UNC-Grewen 2	Prisma	b=0, b=1500, b=3000	79	3222	89.2	1.5x1.5x1.5
13	Northwestern-PHBP	Prisma	b=0, b=1500, b=3000	79	3474	89.2	1.5x1.5x1.5
14	Harvard	TIM Trio	b=0, b=1000	60	4600	89	2x2x2
15	Northwestern-W2W	Prisma	b=0, b=1500, b=3000	79	3474	89.2	1.5x1.5x1.5
16	Denver-Care	Skyra	b=300, b=300, b=800,b=2000	104	6100	60	2x2x2
17	Denver-PP	Prisma	b=0, b=1500,b=3000	98	3222	89.2	1.5x1.5x1.5
18	Rhode Island	TIM Trio	b=0, b=800, b=2500	80	2700	76	2x2x2
		TIM Trio	b=700, b=1500, b=2000	90	7500	104	2.5x2.5x2.5
		Prisma	b=700, b=1500, b=2000	90	7500	104	2.5x2.5x2.5
19	Rochester/Pitt	Skyra	b=0, b=1500,b=3000	79	3222	89.2	1.5x1.5x1.5

Functional connectivity (rsfMRI): We will adapt an established ENIGMA rsfMRI pipeline to define network specific ROIs for functional connectivity assessment(31) in infants and young children. This pipeline differs from others in 2 important respects that make it ideal for harmonizing data across sites: 1) it does not require a structural T1-weighted (T1w) brain MRI scan to regress out nuisance signals from ventricles and white-matter, or for anatomical registration to a common atlas space. Rather, it uses a deformable template created from 1,100 individual T1w images(32), which incorporates spatial distortion patterns. This approach eliminates technical variation that might arise from site-related differences in T1w data quality and co-registration biases that could influence rsfMRI phenotypes. 2) It uses a noise reduction technique based on Marchenko–Pastur Principal Component Analysis (MP-PCA) to improve signal-to-noise ratio (SNR), thus reducing methodological variance across sites(31). Because subject motion can alter time courses of resting state data(33), the pipeline incorporates rigorous motion correction and motion censoring ("scrubbing")(34). To adapt this pipeline for use in infants and young children, we will incorporate brain template and functional atlas developed for infant data into the analytical workflow and merge it with adult template(35). We will calculate ROI-ROI connectivity estimates and use these to extract measures of functional connectivity within Smith's 9 cortical networks, together with system-level efficiency measures based on graph theoretical calculations(36,37), for use in subsequent analyses.

Supplementary Table 6: Resting State fMRI Acquisition Parameters for each ORIGINs site

Cohort	Site	Platform	Scan type	TR	TE	Resolution
1	UNC-EBDS	Allegra	T2*-weighted EPI	2000	32	4x4x4
		TIM Trio	T2*-weighted EPI	2000	32	4x4x4
		Prisma	T2*-weighted EPI	800	37	2x2x2
2	Singapore	Skyra	T2*-weighted EPI	2660	27	3x3x3
3	IBIS	TIM Trio	T2*-weighted EPI	2500	27	4x4x4
4	UNC-Grewen 1	Allegra	T2*-weighted EPI	2000	32	4x4x4
		TIM Trio	T2*-weighted EPI	2000	32	4x4x4
5	UCI	TIM Trio	T2*-weighted EPI	2000	30	3.4x3.4x4
6	MPI	TIM Trio	T2*-weighted EPI	2000	30	3x3x3
7	UNC-MAP	Allegra	T2*-weighted EPI	2000	32	4x4x4
		TIM Trio	T2*-weighted EPI	2000	32	4x4x4
8	UNC-GMIA	TIM Trio	T2*-weighted EPI	2460	32	3x3x3
		Prisma	T2*-weighted EPI	800	37	2x2x2
9	dHCP	Achieva	multiband (MB) 9x accelerated echo-planar imaging	392	38	2.15x2.15x2.15
10	BCP (UNC/UMN)	Prisma	T2*-weighted EPI	800	37	2x2x2
11	DCHS Cape Town	Allegra	T2*-weighted EPI	2000	30	4.0x2.5x2.5
		Skyra	T2*-weighted EPI	2000	30	4.0x2.5x2.5
12	UNC-Grewen 2	Prisma	T2*-weighted EPI	800	37	2x2x2
13	Northwestern-PHBP	Prisma	T2*-weighted EPI	800	37	2x2x2
14	Harvard	TIM Trio	T2*-weighted EPI	3000	30	3x3x3
			Simultaneous multi-slice EPI	950	30	3x3x3
15	Northwestern-W2W	Prisma	T2*-weighted EPI	800	37	2x2x2
16	Denver-Care	Skyra	T2*-weighted EPI	2000	28	3.3x3.3x4
17	Denver-PP	Prisma	T2*-weighted EPI	720	37	2x2x2
18	Rhode Island	TIM Trio	T2*-weighted EPI	2500	34	3x3x3
19	Rochester/Pitt	Skyra	T2*-weighted EPI	720	37	2x2x2

Data Analysis Plans: For each neuroimaging measure we will fit a longitudinal model to capture change over time as in Figure 3. We anticipate that different imaging measures will exhibit different patterns of change We will examine a range of models including linear, quadratic, Gompertz, logistic, and non-parametric models such as splines, and assess via graphical displays and formal model comparison (e.g., based on AIC) which functional form is best. Model parameters (such as intercept and asymptote and predicted values at specific ages) will serve as phenotypes for our genetic analyses.

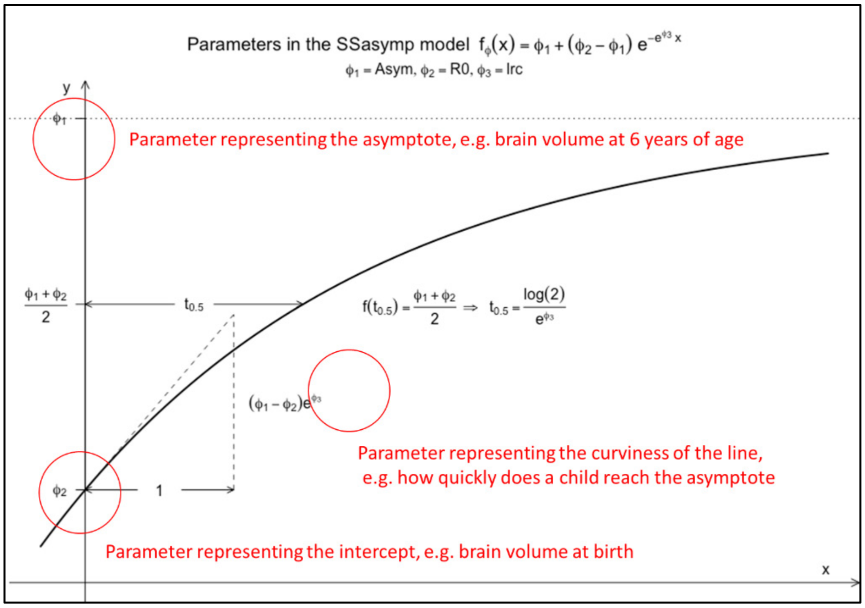

Supplementary Figure 1: Random regression growth curve model to describe the development of an imaging measure.

We refer to these as developmental imaging phenotypes or DIPs. We will use GBLUP models to estimate proportion of variance of each DIP explained by common SNPs (aka SNP-heritability) and to dissect phenotypic associations between DIPs into genetic and non-genetic components (i.e., genetic and environmental correlations). We will use plink to perform multivariate GWA analyses on DIPs. Because the number of traits we will scan is large, we will first cluster traits

according to their (co)variance patterns and perform multi-trait GWA within each phenotype-clusters. We will consider possible sex x SNP interaction effects in additional analyses. Our GWAS Discovery set will consist of data from all cohorts except Rhode Island (n=1,070 kids), Rochester (n=570 kids), and University of Denver Prenatal Pathways (PP, n=200); we will leave this data out for validation. We will use GWA results published by the Psychiatric Genetic Consortium (PGC)(38) to identify DIPs associated with transdiagnostic and disorder-specific risk. Finally, we will test if genetically influenced DIPs predict parent-report measures of impulsivity, anxiety, and aggressive behavior using penalized canonical correlation analysis (CCA).

Challenges and limitations: The different cohorts involved in this group have different study designs including cross sectional, longitudinal, and accelerated longitudinal designs. They have also been genotyped with different platforms and used different scanners and acquisition protocols. Thus, one of the primary challenges of this effort is the harmonization of data across sites. We have already discussed our approach to the harmonization of genetic data. For neuroimaging data, we will address cohort differences via 4 approaches. 1) To reduce variability and potential site bias, we will send all imaging data to a central site for uniform processing and quality control. 2) We will take account of different acquisition parameters in our image analyses as described previously. For example, we will adjust for site-differences in structural MRI resolution by up-or down sampling into 1x1x1mm3isotropic resolution. 3) We note that there are limitations of MRI in studying changes at the early postnatal age and imaging related factors of variance across this early development as biological processes, particularly myelination in infancy, change the interpretation of measurements derived from MR imaging. Technical choices like image resolution also affect measurements differently as brain size varies tremendously from neonate stage to 2 years of age. We use age-appropriate atlases to minimize misspecification of segmented data.

These technical limitations will be considered when interpreting the developmental trajectories that we derive from our MRI data. 4) In our statistical analyses, we will adjust for cohort effects and model site-specific variances by methods similar to those used in ComBat (39), an established batch-effect correction approach that has been used successfully to harmonize cortical thickness metrics, fractional anisotropy maps, and connectivity measures derived from fMRI data (17,18,40–42). 5) In additional analyses we will identify potential site effects, via cross-validation (successively omitting each cohort), and by stratifying analyses by variables that differ across sites, such as prematurity and ancestry. The project aims to obtain ~6100 genomes from infants and young children, imaging data from ~5100 children, and behavioral assessments data from ~ 3800 children. Supplementary table 7 shows details on the varying scan ages and behavioral data ages from the different sites. While it is a limitation that not all sites acquired behavioral data, our planned behavioral analyses are appropriately powered.

Supplementary Table 7: Scan ages and behavioral assessment ages in each cohort

Cohort	Scan Ages	Behavioral Data (Ages)
1	1m, 12m, 24m, 48m, 72m	BASC-2 (48m, 72m)
2	Birth, 54m, 72m	CBCL (24m, 48m)
3	3m, 6m, 9m, 12m, 15m, 24m, 36m	CBCL (36m)
4	1m, 12m, 24m	
5	1m, 12m, 24m	CBCL (18m,24m)
6	37m to 82m	
7	1m	
8	1mand 12m	
9	birth to 1m	CBCL (18m)
10	birth to 60m	CBCL (18m-60m)
11	1m, 24m	CBCL (24m, 42m,60m)
12	1m, 3m, 12m	
13	1m, 6m, 12m	
14	Cohort 1: 2-18m, 60m, 72m, 84m<br>Cohort 2: 2-12m, 14-24m, 60m	
15	12m, 36m	
16	1m	
17	1m	CBCL (6m,12m)
18	birth to 60m	CBCL (18m-72m),<br>BASC-2 (24m-72m)
19	1m, 12m, 48m	BASC-2 (36)

Data Availability: NIMH grant R01 MH123716-01A1 supports secondary analysis of existing genetic data, generation of new genetic data from existing biosamples, and collection and genotyping of new biosamples. In accordance with NIMH data sharing policies, clinical and demographic covariate data, CBCL data, genetic data, and extracted neuroimaging data from children in the latter two categories will be accessible through the NIMH Data Archive (NDA # 3905). This includes children in cohorts 3,4,7,8,10,12,13,14,15,16,17,19 and a subset of cohort 1 (supplementary table 1). Investigators interested in further information on ORIGINs dataset access and sharing can contact the principal investigator (PI) Rebecca Knickmeyer (knickmey@msu.edu). We welcome researchers involved in pediatric imaging genetics and brain-behavior associations to join us in understanding how the genetic variants associated with psychiatric disorders influence early neurodevelopment and the establishment of brain circuitry. Please contact the PI for additional information. The team also encourages domestic and international researchers for collaboration and secondary data analysis projects from this dataset. Secondary data analysis proposals must be approved by each participating cohort and additional data use agreements may be necessary to ensure all sharing is in keeping with participant consents.

We note that some cohorts that will be included in the ORIGINs dataset have already released or will release imaging, behavioral, and/or genetic data that can be accessed without going through ORIGINs. Imaging and behavioral data for twins in Cohort 1 is available through NDA #1974 and NDA #2384 and for singletons via NDA #4314. Genetic data from a subset of Cohort 1 subjects, along with some extracted imaging phenotypes, can be accessed via dbGaP, accession number phs001122.v1.p1. Imaging and behavioral data and some genetic data from Cohort 3 is available via NDA #19 and NDA #2027. Imaging data for Cohort 5 is available via NDA #1890. Information about data releases for Cohort 9 can be found at http://www.developingconnectome.org/. Imaging,

behavioral, and genetic data for Cohort 9 will also be accessible through NDA (Permission Group Developing Human Connectome Project (dHCP)). Imaging and behavioral data for Cohort 10 is available via NDA #2848. Imaging and behavioral data from Cohort 15 will be available through NDA #3422. Imaging and behavioral data for Cohort 16 will be available through NDA #2685.

Software sharing: All MRI processing is based on publicly available tools and atlases. In addition, we will share all our processing scripts, as well as all employed age-specific atlases via github. As these scripts directly operate on data stored in BIDS format, these can be straightforwardly used by any interested researcher aiming to process their own data in the same fashion. As part of statistical analysis, we commit to share any software developed in the project as open source through GitHub or similar platforms (e.g., CRAN).

Supplementary References

1. Xia K, Zhang J, Ahn M, Jha S, Crowley JJ, Szatkiewicz J, *et al.* (2017): Genome-wide association analysis identifies common variants influencing infant brain volumes. *Transl Psychiatry* 7: e1188–e1188.

2. Soh SE, Chong YS, Kwek K, Saw SM, Meaney MJ, Gluckman PD, *et al.* (2014): Insights from the Growing Up in Singapore Towards Healthy Outcomes (GUSTO) Cohort Study. *Ann Nutr Metab* 64: 218–225.

3. Czamara D, Eraslan G, Page CM, Lahti J, Lahti-Pulkkinen M, Hämäläinen E, *et al.* (2019): Integrated analysis of environmental and genetic influences on cord blood DNA methylation in new-borns. *Nature Communications 2019 10:1* 10: 1–18.

4. Zar HJ, Barnett W, Myer L, Stein DJ, Nicol MP (2015): Investigating the early-life determinants of illness in Africa: the Drakenstein Child Health Study. *Thorax* 70: 592–594.

5. Stein DJ, Koen N, Donald KA, Adnams CM, Koopowitz S, Lund C, *et al.* (2015): Investigating the psychosocial determinants of child health in Africa: the Drakenstein Child Health Study. *J Neurosci Methods* 252: 27.

6. Tustison NJ, Avants BB, Cook PA, Zheng Y, Egan A, Yushkevich PA, Gee JC (2010): N4ITK: improved N3 bias correction. *IEEE Trans Med Imaging* 29: 1310–1320.

7. Fonov V, Evans AC, Botteron K, Almli CR, McKinstry RC, Collins DL (2011): Unbiased average age-appropriate atlases for pediatric studies. *Neuroimage* 54: 313–327.

8. Hazlett C, Sylvain Gouttard al, Styner M, Joshi S, Smith RG, Cody Hazlett H, *et al.* (2007): Subcortical structure segmentation using probabilistic atlas priors. *https://doi.org/101117/12708626* 6512: 862–872.

9. Cherel M, Budin F, Prastawa M, Gerig G, Lee K, Buss C, *et al.* (2015): Automatic Tissue Segmentation of Neonate Brain MR Images with Subject-specific Atlases. *Proc SPIE Int Soc Opt Eng* 9413: 941311.

10. Swanson MR, Shen MD, Wolff JJ, Elison JT, Emerson RW, Styner MA, *et al.* (2017): Subcortical Brain and Behavior Phenotypes Differentiate Infants with Autism versus Language Delay. *Biol Psychiatry Cogn Neurosci Neuroimaging* 2: 664.

11. Kim H, Lepage C, Maheshwary R, Jeon S, Evans AC, Hess CP, *et al.* (2016): NEOCIVET: Towards accurate morphometry of neonatal gyrification and clinical applications in preterm newborns. *Neuroimage* 138: 28–42.

12. Kim SH, Lyu I, Fonov V, Vachet C, Hazlett HC, Smith RG, *et al.* (2016): Development of cortical shape in the human brain from 6 to 24months of age via a novel measure of shape complexity. *Neuroimage* 135: 163–176.

13. Destrieux C, Fischl B, Dale A, Halgren E (2010): Automatic parcellation of human cortical gyri and sulci using standard anatomical nomenclature. *Neuroimage* 53: 1.

14. Farzinfar M, Li Y, Verde AR, Oguz I, Gerig G, Styner MA (2013): DTI Quality Control Assessment via Error Estimation From Monte Carlo Simulations. *Proc SPIE Int Soc Opt Eng* 8669: 86692C.

15. Jones DK, Horsfield MA, Simmons A (1999): Optimal strategies for measuring diffusion in anisotropic systems by magnetic resonance imaging. *Magn Reson Med* 42: 515–525.

16. Jones DK, Knösche TR, Turner R (2013): White matter integrity, fiber count, and other fallacies: The do's and don'ts of diffusion MRI. *Neuroimage* 73: 239–254.

17. Radua J, Vieta E, Shinohara R, Kochunov P, Quidé Y, Green MJ, *et al.* (2020): Increased power by harmonizing structural MRI site differences with the ComBat batch adjustment method in ENIGMA. *Neuroimage* 218. https://doi.org/10.1016/j.neuroimage.2020.116956

18. Fortin JP, Parker D, Tunç B, Watanabe T, Elliott MA, Ruparel K, *et al.* (2017): Harmonization of multi-site diffusion tensor imaging data. *Neuroimage* 161: 149–170.

19. Johnson WE, Li C, Rabinovic A (2007): Adjusting batch effects in microarray expression data using empirical Bayes methods. *Biostatistics* 8: 118–127.

20. Basser PJ, Jones DK (2002): Diffusion-tensor MRI: theory, experimental design and data analysis - a technical review. *NMR Biomed* 15: 456–467.

21. Seung JL, Steiner RJ, Yang Y, Sarah JS, Michael CN, Martin AS, *et al.* (2017): Common and heritable components of white matter microstructure predict cognitive function at 1 and 2 y. *Proc Natl Acad Sci U S A* 114: 148–153.

22. Oguz I, Farzinfar M, Matsui J, Budin F, Liu Z, Gerig G, *et al.* (2014): DTIPrep: Quality control of diffusion-weighted images. *Front Neuroinform* 8: 4.

23. Jenkinson M, Beckmann CF, Behrens TEJ, Woolrich MW, Smith SM (2012): FSL. *Neuroimage* 62: 782–790.

24. Andersson JLR, Graham MS, Zsoldos E, Sotiropoulos SN (2016): Incorporating outlier detection and replacement into a non-parametric framework for movement and distortion correction of diffusion MR images. *Neuroimage* 141: 556–572.

25. Verde AR, Berger J-B, Gupta A, Farzinfar M, Kaiser A, Chanon VW, *et al.* (2013): UNC-Utah NA-MIC DTI framework: atlas based fiber tract analysis with application to a study of nicotine smoking addiction. *Medical Imaging 2013: Image Processing*, vol. 8669 8669: 86692D.

26. Goodlett CB, Fletcher PT, Gilmore JH, Gerig G (2009): Group analysis of DTI fiber tract statistics with application to neurodevelopment. *Neuroimage* 45: S133.

27. Verde AR, Budin F, Berger JB, Gupta A, Farzinfar M, Kaiser A, *et al.* (2014): UNC-Utah NA-MIC framework for DTI fiber tract analysis. *Front Neuroinform* 7. https://doi.org/10.3389/fninf.2013.00051

28. Zhu H, Kong L, Li R, Styner M, Gerig G, Lin W, Gilmore JH (2011): FADTTS: Functional Analysis of Diffusion Tensor Tract Statistics. *Neuroimage* 56: 1412.

29. Zhu H, Styner M, Tang N, Liu Z, Lin W, Gilmore JH (2010): FRATS: Functional Regression Analysis of DTI Tract Statistics. *IEEE Trans Med Imaging* 29: 1039.

30. Yuan Y, Gilmore JH, Geng X, Martin S, Chen K, Wang J ling, Zhu H (2014): FMEM: functional mixed effects modeling for the analysis of longitudinal white matter Tract data. *Neuroimage* 84: 753–764.

31. Adhikari BM, Jahanshad N, Shukla D, Glahn DC, Blangero J, Fox PT, *et al.* (2018): Comparison of heritability estimates on resting state fMRI connectivity phenotypes using the ENIGMA analysis pipeline. *Hum Brain Mapp* 39: 4893.

32. Adhikari BM, Jahanshad N, Shukla D, Turner J, Grotegerd D, Dannlowski U, *et al.* (2019): A resting state fMRI analysis pipeline for pooling inference across diverse cohorts: an ENIGMA rs-fMRI protocol. *Brain Imaging Behav* 13: 1453–1467.

33. Power JD, Barnes KA, Snyder AZ, Schlaggar BL, Petersen SE (2012): Spurious but systematic correlations in functional connectivity MRI networks arise from subject motion. *Neuroimage* 59: 2142.

34. Power JD, Mitra A, Laumann TO, Snyder AZ, Schlaggar BL, Petersen SE (2014): Methods to detect, characterize, and remove motion artifact in resting state fMRI. *Neuroimage* 84: 320–341.

35. Shi F, Salzwedel AP, Lin W, Gilmore JH, Gao W (2018): Functional Brain Parcellations of the Infant Brain and the Associated Developmental Trends. *Cerebral Cortex (New York, NY)* 28: 1358.

36. Gao W, Gilmore JH, Giovanello KS, Smith JK, Shen D, Zhu H, Lin W (2011): Temporal and Spatial Evolution of Brain Network Topology during the First Two Years of Life. *PLoS One* 6: e25278.

37. Rubinov M, Sporns O (2010): Complex network measures of brain connectivity: uses and interpretations. *Neuroimage* 52: 1059–1069.

38. Lee PH, Anttila V, Won H, Feng YCA, Rosenthal J, Zhu Z, *et al.* (2019): Genomic Relationships, Novel Loci, and Pleiotropic Mechanisms across Eight Psychiatric Disorders. *Cell* 179: 1469-1482.e11.

39. Chen C, Grennan K, Badner J, Zhang D, Gershon E, Jin L, Liu C (2011): Removing Batch Effects in Analysis of Expression Microarray Data: An Evaluation of Six Batch Adjustment Methods. *PLoS One* 6: e17238.

40. Fortin JP, Cullen N, Sheline YI, Taylor WD, Aselcioglu I, Cook PA, *et al.* (2018): Harmonization of cortical thickness measurements across scanners and sites. *Neuroimage* 167: 104–120.

41. Pomponio R, Erus G, Habes M, Doshi J, Srinivasan D, Mamourian E, *et al.* (2020): Harmonization of large MRI datasets for the analysis of brain imaging patterns throughout the lifespan. *Neuroimage* 208. https://doi.org/10.1016/j.neuroimage.2019.116450

42. Yu M, Linn KA, Cook PA, Phillips ML, McInnis M, Fava M, *et al.* (2018): Statistical harmonization corrects site effects in functional connectivity measurements from multi-site fMRI data. *Hum Brain Mapp* 39: 4213–4227.
